# Associations between residence at birth and mental health disorders: a spatial analysis of retrospective cohort data

**DOI:** 10.1186/s12889-015-2011-z

**Published:** 2015-07-21

**Authors:** Kate Hoffman, Ann Aschengrau, Thomas F. Webster, Scott M. Bartell, Verónica M. Vieira

**Affiliations:** Nicholas School of the Environment, Duke University, Durham, NC USA; Boston University School of Public Health, Boston, MA 02118 USA; Program in Public Health, University of California, Irvine, 653 E. Peltason Drive, AIRB 2042, Irvine, CA 92697 USA

**Keywords:** Epidemiology, Bipolar disorder, Depression, Generalized additive models, Post-traumatic stress disorder, Spatial analysis

## Abstract

**Background:**

Mental health disorders impact approximately one in four US adults. While their causes are likely multifactorial, prior research has linked the risk of certain mental health disorders to prenatal and early childhood environmental exposures, motivating a spatial analysis to determine whether risk varies by birth location.

**Methods:**

We investigated the spatial associations between residence at birth and odds of depression, bipolar disorder, and post-traumatic stress disorder (PTSD) in a retrospective cohort (Cape Cod, Massachusetts, 1969–1983) using generalized additive models to simultaneously smooth location and adjust for confounders. Birth location served as a surrogate for prenatal exposure to the combination of social and environmental factors related to the development of mental illness. We predicted crude and adjusted odds ratios (aOR) for each outcome across the study area. The results were mapped to identify areas of increased risk.

**Results:**

We observed spatial variation in the crude odds ratios of depression that was still present even after accounting for spatial confounding due to geographic differences in the distribution of known risk factors (aOR range: 0.61–3.07, *P* = 0.03). Similar geographic patterns were seen for the crude odds of PTSD; however, these patterns were no longer present in the adjusted analysis (aOR range: 0.49–1.36, *P* = 0.79), with family history of mental illness most notably influencing the geographic patterns. Analyses of the odds of bipolar disorder did not show any meaningful spatial variation (aOR range: 0.58–1.17, *P* = 0.82).

**Conclusion:**

Spatial associations exist between residence at birth and odds of PTSD and depression, but much of this variation can be explained by the geographic distributions of available risk factors. However, these risk factors did not account for all the variation observed with depression, suggesting that other social and environmental factors within our study area need further investigation.

## Background

Annually, one in four US adults suffers from a diagnosable mental illness such as depression, bipolar disorder, or post-traumatic stress disorder (PTSD) [1, 2]. Their causes are likely multifactorial; however, research indicates that adverse prenatal events increase the risk of mental disorders [3]. For example, exposure to maternal stress, malnutrition, and infection in early gestation have been shown to increase the risk of developing mental illness as an adult [3–10]. While maternal alcohol and tobacco use during pregnancy have also been associated with increased risk of psychiatric disorders [11–16], relatively little is known about the potential for other prenatal exposures, including environmental toxicants, to impact mental health.

Investigations of prenatal exposures and their impact on mental health are complicated by the lengthy period between the exposure and onset of symptoms, making exposure assessment difficult. Residential information during prenatal development, however, provides a useful tool in assessing social and environmental exposures that are geographically distributed. A recent study by Aschengrau et al. [17] used residential location and water distribution information to investigate the relationship between prenatal and early childhood exposures to tetrachloroethylene (PCE) contaminated drinking water and mental health disorders on Cape Cod, Massachusetts. Children exposed to PCE prenatally and during early-life were at increased risk of PTSD and bipolar disorder as an adult [17]. These results motivated the current spatial analyses whose goal was to determine whether residence at birth was associated with mental health disorders.

Exposures to many environmental contaminants vary geographically on Cape Cod, including air and water pollution associated with the Massachusetts Military Reservation, pesticide applications to cranberry bogs, and particulate air pollution from a large electric power plant [18–23]. Cape Cod communities are socioeconomically diverse, with variable rates of poverty, educational attainment, income, and unemployment, both over time and geographically [24]. To determine whether prenatal exposures to these and other geographically distributed factors in Cape Cod impact the development of mental illness, we investigate geographic birth location as a surrogate for exposure to a mix of social and environmental exposures. We assessed differences in the spatial distribution of mental health outcomes on Cape Cod with three objectives: 1) to describe geographic variation in mental health outcomes based on the residential location at birth; 2) to identify whether these spatial patterns were due to geographic variation in many known risk factors for mental health; and 3) to generate etiologic hypotheses for future epidemiologic investigations if spatial variation persisted after adjustment for spatial confounding. Using individual-level data collected as part of a larger retrospective cohort study [17], we investigated the spatial associations between residence at birth and odds of depression, bipolar/manic-depressive disorder, and PTSD.

## Methods

### Study population

Data were collected from a series of retrospective cohort studies investigating potential health impacts associated with prenatal and early-life exposures to PCE-contaminated drinking water on Cape Cod, Massachusetts (as described in [17, 25]). Eligible subjects were born to married mothers between 1969 and 1983. Although the original cohort included children born in eight Cape Cod towns, the current analyses were restricted to children born to families living in the spatially contiguous upper Cape Cod towns of Barnstable, Bourne, Falmouth, Mashpee, or Sandwich at the time of the child’s birth. In the initial phases of the study, birth certificates and questionnaires completed by mothers (or fathers, if mothers were unavailable) were used to gather information on the prenatal period and early childhood. These data were used previously to investigate geographic differences in prenatal and early childhood development as well as the impacts of PCE exposure [26–31]. To obtain information about adolescent and adult health outcomes and behaviors, offspring were followed-up during 2006–2008, when they were 23 to 39 years of age [17, 25]. Approximately 33 % of the original eligible cohort participated in the follow-up study [17, 25]; follow-up participants were similar to non-participants with respect to race, age, birth order, PCE exposure status, birth weight, and gestational duration but differed by maternal education and sex, with female offspring and those of college educated mothers being more likely to participate in the follow-up study [32, 33]. The original retrospective cohort studies focused on prenatal and early-life exposures, so information on many adverse events that occurred later in life such as job loss were not collected. Our current work, as well as all previous phases of the study, was reviewed and approved by the Massachusetts Department of Public Health, and the Institutional Review Boards at the Boston University Medical Center and the University of California at Irvine.

### Residential history

Mothers provided a residential history including the address at birth for all participants in the initial study. These addresses were geocoded to longitude and latitude using ArcGIS (ArcGIS version 8.1, ESRI; Redlands, CA) as described previously [17, 25]. In the original cohort, approximately 95 % of the reported addresses were geocoded. For remaining addresses, information was insufficient for geocoding, and these participants were excluded from analyses.

### Mental health outcomes

Study participants completed a self-administered questionnaire which included information on demographic characteristics including age, race, ethnicity, marital status, educational attainment, and occupation. The survey also included questions on mental illness. In particular, participants were also asked if a doctor or health care provider had ever said that they had depression, bipolar disorder, manic-depressive disorder, or PTSD, and whether any first degree relatives had been diagnosed with these conditions. Of a potential 1,278 study participants, 1,256 (98.3 %), provided mental health and address information sufficient for inclusion in the spatial analyses.

### Spatial analyses

The spatial methods applied in these analyses have been described in detail in Webster et al. [34] and Bliss et al. [35]. Briefly, we estimated the log odds of each mental health outcome using GAMs, an extension of linear regression models that can be used to analyze binary data and can accommodate both parametric and non-parametric model components [34, 36, 37]. For non-parametric model terms, GAMs replace the traditional beta coefficient from an ordinary logistic regression with a smooth term. In spatial analyses we applied a bivariate smooth to latitude and longitude coordinates and included all other covariates as parametric terms [34, 36]. We used a locally weighted regression smoother (loess) in the analyses. The loess smoother utilizes information from nearby data points (weighting information based on its distance from the prediction point) to predict the log odds of each outcome. The optimal amount of smoothing, referred to as the optimal span size, describes the region or neighborhood from which data are drawn for prediction and is based on the percentage of data points in the neighborhood. Choice of span size is a trade-off between bias and variability. A larger span size with more data included results in a flatter surface with lower variability but increased bias in predicted log odds, while a smaller span size (less data included) results in higher variability and lower bias in comparison. We determined the optimal span size by minimizing Akaike’s Information Criterion [34, 36].

We predicted the log odds for each mental health outcome using a grid of points that ranged across the latitude and longitude coordinates of participants’ birth addresses. We excluded grid points where participants did not live (*e.g.,* conservation land), resulting in a grid of approximately 3,500 points which covered upper Cape Cod, MA. Log odds were converted to odds ratios using the entire study area as the referent group by dividing the individual point log odds by the log odds from a regression model with just the covariates [34].

We tested the null hypothesis that the odds of each mental health outcome did not depend on the geographic location at birth using permutation tests [34, 38]. The deviance statistic was calculated for each new GAM model fit to a dataset where location was permuted but case status and covariates were held fixed. We ran 999 unconditional permutations [38], determining the optimal span for each GAM model [35], and ranked the deviances of all the models to compute the global statistic. Local point-wise permutation tests were conducted if the global statistic indicated that residential location was a statistically significant predictor of an outcome (*p* < 0.05). Log odds in the upper and lower 2.5 % of the distribution were denoted on the map as areas of significantly increased or decreased odds using black contour bands. Results were mapped using a continuous color scheme (dark red to dark blue) and a constant scale range for each outcome (ArcMap, version 10.0, ESRI; Redlands, CA). Spatial analyses were conducted in R (version 2.12.02; Vienna Austria) using the gam (version 1.09) and MapGam (version 0.7-0) packages [39, 40].

### Spatial confounding

Potential confounders were identified through a review of the scientific literature on mental illness and included sex; year of birth; low birth weight (<2500 g *vs.* ≥2500 g), preterm delivery (<37 weeks *vs.* ≥37 weeks); family history of mental health illness; socioeconomic status at birth (father’s occupation and mother’s educational attainment); parental age; maternal cigarette smoking and alcoholic beverage consumption during pregnancy; pre/postnatal exposure to PCE; and personal history of attention deficit/attention deficit hyperactivity disorders, learning difficulties, or chronic illness [11, 17, 32, 33, 41–45]. We investigated spatial confounding by these variables (*i.e.,* difference in the geographic distribution of outcome risk factors) using several criteria. First, we investigated geographic variability in the distribution of potential confounders to determine if the variables were associated with location. We then conducted spatial analyses adjusting for each potential confounder individually to determine which variables were the strongest risk factors by comparing changes in spatial patterns of mental health outcome odds between the crude and adjusted models. Finally, we included these variables together in the model, adding each in order of their individual importance, and compared the crude results to the adjusted model to determine if the additional confounders changed the surface of odds ratio. Based on these criteria, we included sex, year of birth, family history of mental health diagnosis, father’s occupation, maternal educational attainment, maternal smoking during pregnancy, and pre- and postnatal PCE exposure in all fully adjusted models.

Although data were generally complete, information on the family history of mental illness and prenatal exposures was missing for 6 to 20 % of participants. Rather than exclude participants with missing data from analyses, we imputed values for missing covariates using multivariate imputation by fully conditional specification (PROC MI in SAS; version 9.3; SAS Institute Inc., Cary, NC). Values were imputed for all covariates with missing data using information for the larger study cohort (all eight towns) to inform prediction. All analyses were conducted for five imputed datasets. For these datasets, results were nearly identical; as such, we present results of spatial analyses using a single imputed dataset. Additionally, to determine the impact of missing data and the use of imputed data on our results, we also conducted analyses restricted to individuals with complete information for all included covariates.

### Sensitivity analyses

Our study cohort included 124 sibling pairs. In addition to genetic similarities, many of the siblings were born at the same residential address. Including multiple children from the same family in analyses could, therefore, induce clustering of mental illness outcomes as a result of familial factors. To determine the robustness of our results to including multiple children from the same family, we conducted sensitivity analyses which included one randomly selected participant from each family.

Our primary hypothesis is that the residential location at the time of birth, a proxy for the social and environmental factors during the prenatal period, is associated with mental illness. However, because the duration of residence after birth varied considerably in our cohort, we conducted sensitivity analyses to evaluate whether impacts were modified by residential duration. We stratified our cohort into two groups: those remaining at the birth location for 10 years or more and those moving before age 10. Analyses investigating spatial patterns of depression were completed in both strata, but there were too few cases to assess PTSD or bipolar disorder to conduct these stratified analyses.

## Results

**Fig. 1:**
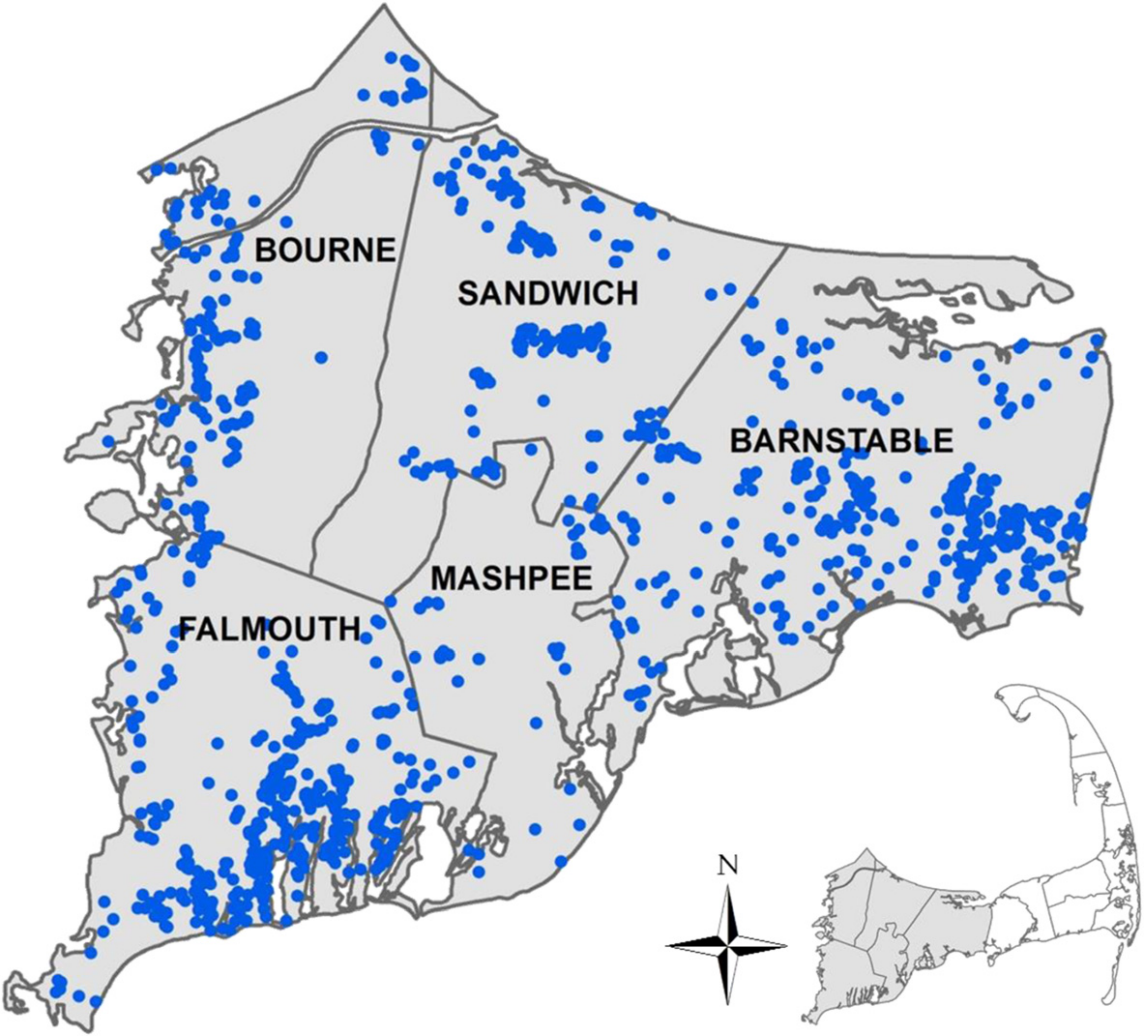
Residential location at the time of birth for 1256 study participants born on Upper Cape Cod, MA from 1969 to 1983. Locations have been altered to preserve confidentiality

Our study population consisted of 1,256 adults born to women living on upper Cape Cod, Massachusetts between 1969 and 1983. At the time of interview, participants were on average in their late 20s (mean age 29.4 ± 3.8 years), college educated (60.0 %), and employed (87.6 %). Table 1 presents the distribution of characteristics included in the final analysis. Participants were more likely to be female (59.8 %) and the vast majority were of white race (98.4 %). Residential locations at birth are displayed in Fig. 1, with the locations altered slightly to protect confidentiality. Overall, 279 (22.2 %) of participants reported a diagnosis of depression, bipolar disorder, or PTSD (46 participants reported multiple mental illness diagnoses). Depression was, by far, the most commonly reported diagnosis, with 17.4 % of participants reporting that they had been told by a doctor or health care professional that they had this diagnosis. Bipolar disorder and PTSD impacted far fewer participants (3.5 and 4.9 %, respectively).

**Table 1:** Distribution of Participants by Selected Characteristics, Cape Cod, Massachusetts, 1969–1983

Characteristic	No. (%)
Total	1256 (100 %)
Sex	
Male	505 (40.2 %)
Female	751 (59.8 %)
Year of birth	
1969–1974	278 (22.9 %)
1975–1980	653 (52.0 %)
1981–1983	325 (25.9 %)
Mother's educational attainment	
High School Graduate or Less	478 (38.1 %)
Some College	382 (30.4 %)
College graduate or More	396 (31.2)
Missing	3 (0.2 %)
Father’s occupation	
White Collar	620 (49.4 %)
Blue Collar	405 (32.2 %)
Other	215 (17.1 %)
Missing	16 (1.3 %)
Maternal cigarette smoking during pregnancy	
No	728 (58.0 %)
Yes	271 (21.6 %)
Missing	257 (20.5 %)
Pre/postnatal PCE exposure	
No	429 (34.2 %)
Yes	749 (59.6 %)
Missing	7 (6.2 %)
Family history of mental illness	
No	696 (55.4 %)
Yes	489 (38.9 %)
Missing	71 (5.7 %)

**Fig. 2:**
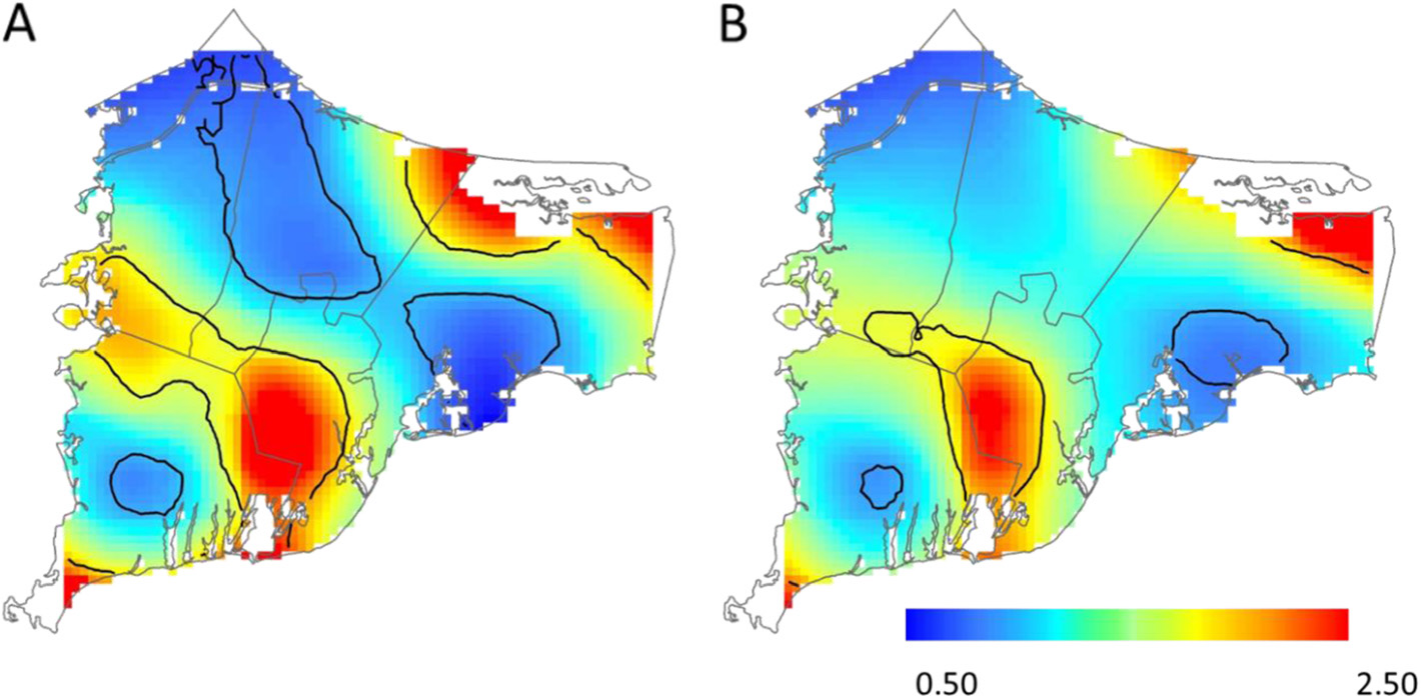
Geographic distribution of depression *vs.* no reported mental illness from analyses using the optimal span size for each model [unadjusted (**a**) and adjusted for sex, year of birth, family history of mental health diagnosis, father’s occupation, mother’s educational attainment, maternal smoking during pregnancy, and pre/postnatal PCE exposure (**b**)]. Black contour bands indicate areas of statistically significant increased or decreased odds of outcomes. The scale includes most, but not all, observed odds ratios

**Fig. 3:**
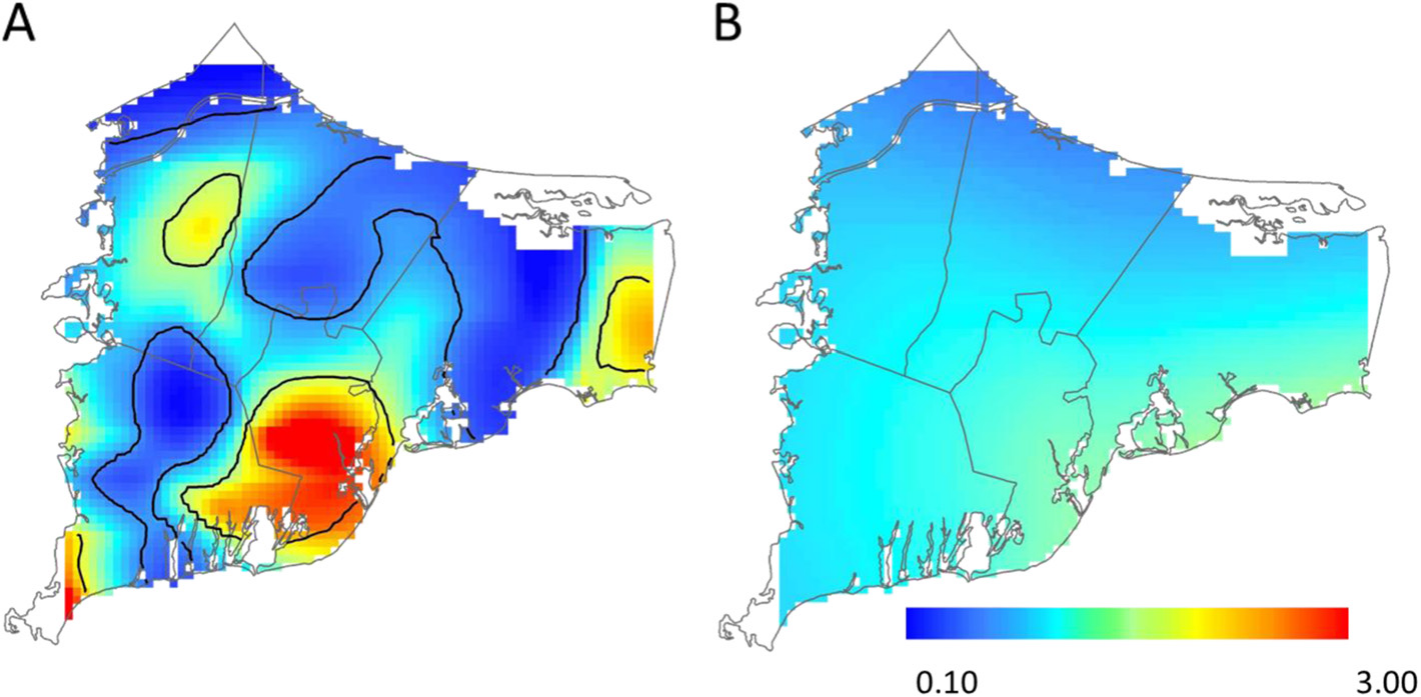
Geographic distribution of PTSD *vs.* no reported mental illness from analyses using the optimal span size for each model [unadjusted (**a**) and adjusted for sex, year of birth, family history of mental health diagnosis, father’s occupation, mother’s educational attainment, maternal smoking during pregnancy, and pre/postnatal PCE exposure (**b**)]. Black contour bands indicate areas of statistically significant increased or decreased odds of outcomes. The scale includes most, but not all, observed odds ratios

**Fig. 4:**
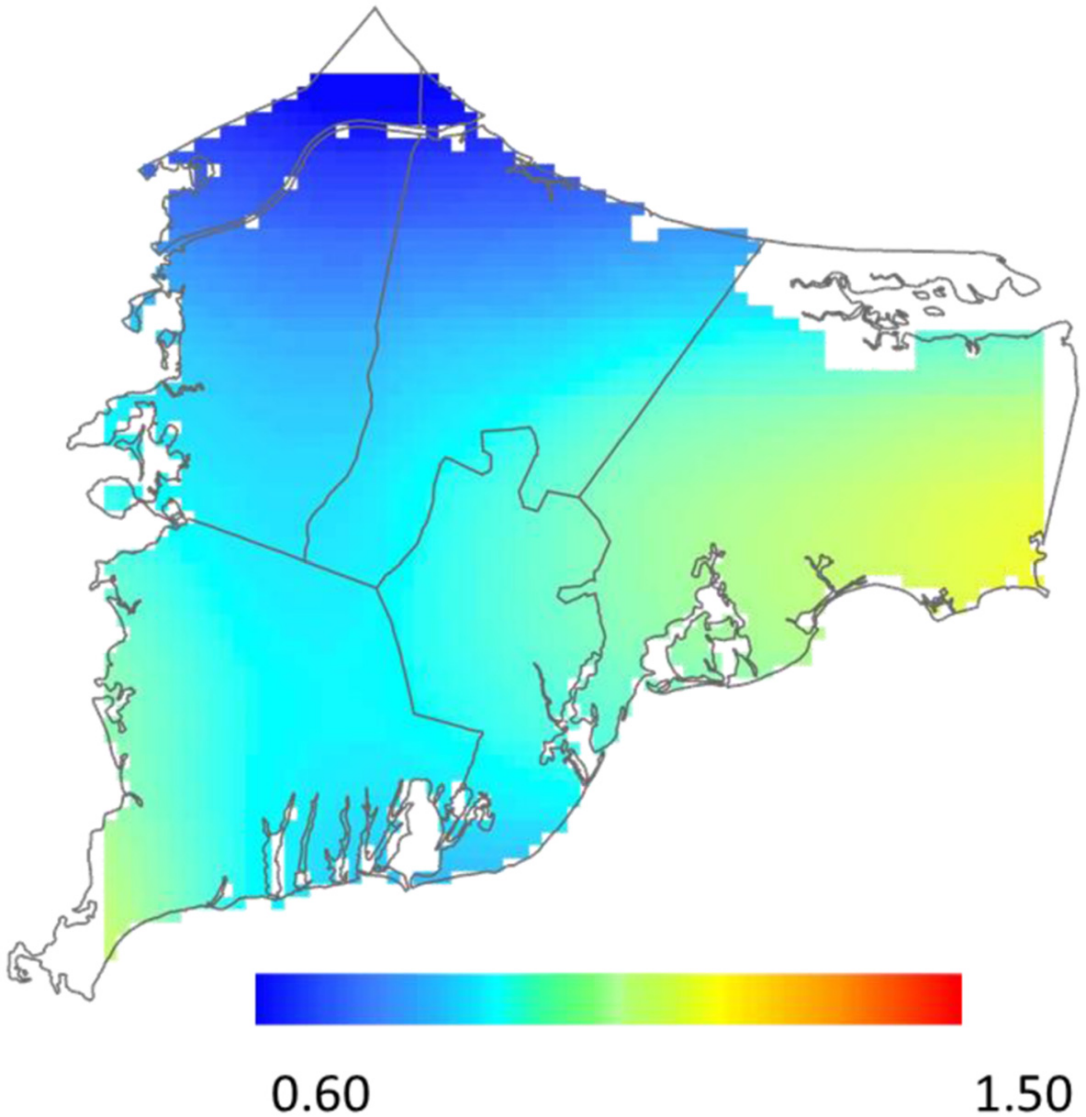
Geographic distribution of bipolar disorder *vs.* no reported mental illness adjusted for sex, year of birth, family history of mental health diagnosis, father’s occupation, mother’s educational attainment, maternal smoking during pregnancy, and pre/postnatal PCE exposure. Results for the unadjusted analysis were very similar (not shown)

Before adjustment for spatial confounding, we observed statistically significant geographic variation in the odds of depression for children born to women living on upper Cape Cod between 1969 and 1983. Children born in Mashpee and portions of northern Barnstable were approximately 1.75 to 2.50 times as likely to be diagnosed with depression compared to children born in the study area as a whole (Table 2; Fig. 2a). Conversely, children born in Bourne, Sandwich, and southern portions of Barnstable had lower odds of depression. Adjusting for spatial confounding slightly decreased the size of areas of increased and decreased odds; however, results continued to suggest spatial variation in the odds of depression, particularly in in areas of Mashpee and Barnstable (Fig. 2b). Prior to adjustment for spatial confounding, we observed a similar pattern for PTSD, with increased odds in Mashpee and portions of Barnstable compared to the study area as a whole. In Mashpee, however, the pattern appeared to be driven by a greater proportion of participants with a family history of mental illness living in the area (Fig. 3a). After adjustment for family history and other confounders, the optimal span size increased from 20 to 95 % of the data and the global deviance statistic, which was statistically significant in the crude model (*P* = 0.01), was no longer significant (*P* = 0.80; Table 2; Fig. 3b). Similarly, as expected with the increased optimal span size, the range of odds ratios was much narrower across the study area following adjustment (adjusted odds ratio (OR) range: 0.49 to 1.36 across the study area compared to crude OR range: 0.07–3.50). In general, after adjustment for spatial confounding, the odds of PTSD were slightly decreased in northern portions of the study area and slightly increased in southern portions. We did not find evidence of spatial variation in the risk of bipolar disorder in either the unadjusted or adjusted models. Like the adjusted PTSD analyses, the odds of bipolar disorder were lower in northern portions of the study area and slightly increased in southern portions of Barnstable (Table 2; Fig. 4).

**Table 2:** Spatial model summaries, Cape Cod, Massachusetts, 1969–1983

Outcome		Unadjusted				Adjusted			
	Cases/Non-cases	Span	*P*-value	OR Range	Figure #	Span	*P*-value	OR Range	Figure #
Mental Illness Diagnosis									
Depression *vs.* No reported mental illness	219/977	0.30	0.02	0.50–2.43	2a	0.35	0.03	0.61–3.07	2b
Post-Traumatic Stress Disorder *vs.* No reported mental illness	62/977	0.20	0.02	0.07–3.50	3a	0.95	0.79	0.49–1.36	3b
Bipolar Disorder *vs.* No reported mental illness	44/977	0.95	0.80	0.63–1.50		0.95	0.82	0.58–1.17	4

When we restricted the analyses to only one randomly selected child from each family, we found that the spatial patterns were nearly identical to those based on multiple children from the same family (results not shown). Similarly, when we restricted analyses to participants with complete data, results were similar to those based on imputed missing data (results not shown). Investigating the odds of depression for those who remained at the birth address for ≥10 years produced very similar results to those conducted in the cohort as a whole. Analyses of participants that moved from their birth address during the first 10 years of life were also quite similar to analyses in the full cohort, suggesting that the residential location at the time of birth is driving these associations (results not shown).

## Discussion

Overall, our results indicate that children born to mothers living in northern Sandwich and Bourne had decreased odds of mental illness. We also observed increased odds of depression in Mashpee which remained significantly elevated even after adjustment for known prenatal risk factors. The residual spatial variation we observed suggests that other risk factors for depression, including prenatal or postnatal socioeconomic status (SES) and environmental factors, may not be equally distributed on upper Cape Cod. For example, post-natal risk factors for mental illness include loss of a job, death of a loved one, and serious illness; these may be geographically clustered and influenced by risk factors other than residence at birth, sex, year of birth, family history of mental health diagnosis, father’s occupation, mother’s educational attainment, maternal smoking during pregnancy, and pre- and postnatal PCE exposure.

The area of Mashpee where we observed an increased odds of depression is south of the Massachusetts Military Reservation. Although our results should not be interpreted as showing an association between the Military Reservation and depression, they do suggest that environmental exposures and community factors in this area may warrant investigation in future epidemiologic studies. Alternatively, although the use of GAMs allowed us to control for many spatial confounders, residual confounding could also explain the observed spatial variation. For example, several confounders (family history of mental health diagnosis, father’s occupation, mother’s educational attainment, and maternal smoking during pregnancy) were characterized using questionnaire responses that may not have been 100 % accurate; misclassification in confounding variables can lead to incomplete adjustment [46] (Savitz and Baron, 1989).

In analyses of PTSD, adjusting for a family history of mental illness resulted in much flatter maps and eliminated an area of increased odds in Mashpee. Adjusting for a family history of mental illness may, however, mask important associations, as the same factors contributing to participant’s mental illness may also have contributed to the mental illness of a relative who is also living in the same area. Unfortunately, we do not have information on the residential history of family members with mental illness needed to disentangle these relationships.

Study participants were born between 1969 and 1983. Although we had a relatively large sample size (*n* = 1,256), we were unable to examine the geographic distribution of mental illness over time because of the relatively small number of cases. Combining residential data over a 14 year period may also have limited our ability to identify areas with short-term increases in risk, reflecting episodic exposures (*e.g.,* prenatal exposure to infectious agents or natural disaster) which have been linked to mental health outcomes in other regions [4, 5, 47, 48].

Our results should also be interpreted in the context of several additional limitations. Importantly, we did not have information on some potentially important postnatal risk factors for mental health, such as military service or job loss [49, 50]. Although these factors are possible effect modifiers for the odds of mental illness with respect to birth location, the extent to which postnatal risk factors could confound associations between residential location at birth and mental illness is unclear. Events occurring after birth cannot affect residential location at birth, indicating that they are not confounders [51]. Some unmeasured postnatal risk factors such as adult residential location, adult SES, and marital status may be on a causal pathway from location at birth to mental illness and therefore should not be included as confounders in our models; any such indirect effects of birth location are implicitly included in our spatial analyses. However, our analyses may be confounded if postnatal risk factors are also affected by a shared cause of residential location at birth that is not explained by the measured pre-natal risk factors. For example, consider participant military service as an unmeasured postnatal risk factor for PTSD. If a parent’s military service influenced both whether the participant entered the military and the location of the birth residence, independently of the effects of all measured pre-natal risk factors included in our models, then parent’s military service is a confounder of the association between residential location and PTSD, acting through the postnatal risk factor of participant military service. We were not able to adjust for participant's or parent's military service in our analyses, nor could we assess the effects of postnatal risk factors on the risk of mental illness. Future studies on birth location and mental illness might benefit from measurement of military service, job loss, and other known or suspected risk factors.

In addition, outcome data were self-reported, potentially resulting in misclassification. We anticipate that this misclassification was non-differential with respect to birth residence; nonetheless it may have limited our ability to detect meaningful associations. Similarly, we used a single birth address as an indicator of community exposures during the entire prenatal period. Families may have moved during this period, however, potentially resulting in exposure misclassification. Postnatal exposures may also be important in the development of mental illness. Because we had residential history information, we were able to assess the influence of birth residence duration on the odds of depression by restricting to participants who lived at the birth residence for different periods of time. Results were similar regardless of whether a participant had moved during early childhood, suggesting that associations may be driven by factors related to the geographic location at the time of birth. However, we were unable to investigate the impacts of residential mobility on the odds of PTSD or bipolar disorder because these diagnoses were reported by too few participants for stratification. Additionally, participation in the follow-up study was low (approximately 33 % of the original eligible cohort participated). It is possible that individuals with mental illness declined to participate at a higher rate, reducing the number of cases in the final study population. However, as reported previously, low participation was not likely to create selection bias as characteristics of participants and non-participants, including age, race, duration of gestation, birth weight, and birth order, were very similar. Residential location at birth was also similar among participants and non-participants [17, 25]. Finally, reported odds ratios are higher than risk ratios due to the prevalence of the disease outcomes, particularly for depression which is common in our study population.

The spatial analyses methods that we used also have some important limitations. GAMs may exhibit biased behavior at the edges of the data, although our work with synthetic data suggests little or no bias when a loess smoother is used [34]. Additionally, to address this issue, we did not predict odds ratios for areas where participants did not live (*e.g.,* conservations lands). We also determined the optimal amount of smoothing by minimizing the AIC [36]; however using a large spans size may obscure interesting spatial variations in disease odds at a smaller scale. Lastly, although our sensitivity analyses indicated that siblings did not influence our results, a generalized additive mixed model (GAMM) framework would be more appropriate. We are currently working on extending and evaluating a two-dimensional loess smooth using GAMMs.

## Conclusions

Taken together, our results suggest an increased risk of depression among children born to pregnant women living in portions of Mashpee and Barnstable between 1969 and 1983. These geographic differences in risk may be due to differences in the underlying distribution of risk factors in these areas, suggesting that additional research may help to identify risk factors for depression that act during the prenatal period.

## Abbreviations

GAM: Generalized additive model
OR: Odds ratio
PCE: Tetrachloroethylene
PTSD: Post-traumatic stress disorder
SES: Socioeconomic status
